# A Race between Tumor Immunoescape and Genome Maintenance Selects for Optimum Levels of (epi)genetic Instability

**DOI:** 10.1371/journal.pcbi.1002370

**Published:** 2012-02-16

**Authors:** Shingo Iwami, Hiroshi Haeno, Franziska Michor

**Affiliations:** 1 PRESTO, Japan Science and Technology Agency, Graduate School of Mathematical Sciences, The University of Tokyo, Institute for Virus Research, Kyoto University, Kyoto, Japan; 2 Department of Biostatistics and Computational Biology, Dana-Farber Cancer Institute, and Department of Biostatistics, Harvard School of Public Health, Boston, Massachusetts, United States of America; University of Nottingham, United States of America

## Abstract

The human immune system functions to provide continuous body-wide surveillance to detect and eliminate foreign agents such as bacteria and viruses as well as the body's own cells that undergo malignant transformation. To counteract this surveillance, tumor cells evolve mechanisms to evade elimination by the immune system; this tumor immunoescape leads to continuous tumor expansion, albeit potentially with a different composition of the tumor cell population (“immunoediting”). Tumor immunoescape and immunoediting are products of an evolutionary process and are hence driven by mutation and selection. Higher mutation rates allow cells to more rapidly acquire new phenotypes that help evade the immune system, but also harbor the risk of an inability to maintain essential genome structure and functions, thereby leading to an error catastrophe. In this paper, we designed a novel mathematical framework, based upon the quasispecies model, to study the effects of tumor immunoediting and the evolution of (epi)genetic instability on the abundance of tumor and immune system cells. We found that there exists an optimum number of tumor variants and an optimum magnitude of mutation rates that maximize tumor progression despite an active immune response. Our findings provide insights into the dynamics of tumorigenesis during immune system attacks and help guide the choice of treatment strategies that best inhibit diverse tumor cell populations.

## Author Summary

Immunologic surveillance is a function of the immune system which serves to constantly monitor the body for microorganisms, foreign tissue, and cancer cells. To evade this surveillance and subsequent elimination, cancer cells evolve strategies to prevent being recognized and killed by immune system cells; one mechanism is to increase the rate at which genetic and/or epigenetic variability is generated. The benefits of an increased variability of cancer cells to counteract immune surveillance, however, stands in contrast to the costs associated with such heightened mutation rates: the risk of an inability to maintain essential genome structure and functions. To study such situations arising in tumorigenesis, we designed a novel mathematical framework of tumor immunosurveillance and the evolution of mutation rates. We then utilized this framework to study how increased mutation rates and immunologic surveillance affect the abundance of tumor and immune system cells. We found that there exists an optimum number of tumor variants and an optimum magnitude of mutation rates that maximize tumor progression despite the presence of actively proliferating and functioning immune system cells. Our study contributes to an understanding of cancer development during immune system attacks and also suggests treatment strategies for heterogeneous tumor cell populations.

## Introduction

In 1909, Paul Ehrlich was the first to propose the idea that the immune system scans for and eliminates nascent transformed cells in the human body [1]. This hypothesis received much interest from both immunologists and cancer researchers and led to experiments with tumors transplanted into mice; these studies suggested the existence of tumor-associated antigens and formed the basis of the idea of immune surveillance [2]. Since these landmark studies in the 1950s, the model of cancer immune surveillance has gained widespread acceptance, and the central role of immune effector cells, such as B, T, and natural killer (NK) cells, have been elucidated [3], [4], [5], [6], [7]. NK cells and CD8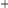 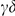 T cells were found to recognize and kill tumor cells through the interaction of specific cell surface receptors with tumor cell ligands [3], [8], [9], [10], [11], [12]. Similarly, CD4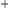 and CD8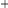 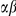 T cells recognize MHC class II and class I molecules on tumor cells, respectively, and B cells produce antibodies against tumor antigens [3], [6], [13]. When the immune system fails to eliminate all tumor cells, then the malignant cell population continues to grow – a phenomenon termed “tumor immunoescape”. The interaction with the immune system, however, may significantly decimate the abundance of tumor cells and select for those phenotypes with relative resistance against immune system attacks. The “cancer immunoediting” hypothesis then predicts that, while one outcome is complete eradication of a developing tumor, another is the generation of a sculpted tumor cell population that either displays reduced immunogenicity [4] or an increased ability to inhibit anti-tumor immune responses [6], [14], [15], [16]. The latter capacity may be imparted via diverse mechanisms [17], [18]: (i) tumor cells can lose their MHC class I molecules, enabling them to evade CTL attacks [19]; (ii) while the immunodominant epitope becomes the main target of immune responses, cells with other phenotypes may continue proliferating in the “shadow” of the dominant clone [20]; (iii) furthermore, tumor cell secretion of immunosuppressive cytokines such as TGF-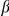 and IL-10 can reduce the efficiency of the immune response [21], and (iv) a modification of death signaling may prevent cells from undergoing apoptosis [22].

Tumor immunoescape is driven by the generation of tumor cell variants [17], [23]. Frequent genetic and epigenetic alterations enable tumor cells to lose MHC class I molecules, produce immunosuppressive cytokines, and generate other phenotypes that are selected to escape immunosurveillance. Although cells with normal rates of accumulating such alterations may also manage to evade the immune response, this process is accelerated by the evolution of genomic instabilities [24], [25]. Genomic instabilities are common in most cancer types [26], and two main categories have been identified: in the majority of tumors, chromosomal instability (CIN) leads to an increased rate of losing or gaining whole chromosomes or parts of chromosomes during cell division [24]; in a smaller fraction of cancers, a mismatch-repair deficiency leads to microsatellite instability (MIN) at the nucleotide level [27]. Similar to genomic instabilities, epigenetic instabilities were also recently found to contribute to tumorigenesis by modulating the production of oncogenic proteins [28].

An increased chance of accumulating (epi)genetic alterations during cell divisions enhances the rate of generating tumor cell variants that may evade the immune response; however, high rates of alterations may in turn lead to an error catastrophe in that a functioning genome cannot be sustained when error-prone replication produces excess damage [29]. The concept of an error catastrophe was first introduced to describe the behavior of RNA viruses [30], and numerous observations about the extinction of such viruses due to excess error have been reported [31], [32], [33]. These findings imply that a mutator phenotype does not serve as an unequivocal benefit for tumor cells, but also harbors a risk of extinction if the extent of variability in the population crosses a threshold. A delicate balance between the cost of a potential error catastrophe and the benefits of outracing the immune response enables tumor cells to survive and expand despite immune system attacks.

Several mathematical models have been designed to provide insights into the dynamics of tumorigenesis under immunosurveillance or an error catastrophe of tumor cells [34], [35], [36], [37], [38], [39], [40], [41]. Most studies of the effects of immunosurveillance on tumor evolution considered a homogeneous population of tumor cells and concentrated on phenomena such as tumor dormancy and immunoescape. Studies of the tumor error catastrophe, in contrast, investigated quasispecies models in simplified situations without an immune response. The dynamics of tumor immunoescape and error thresholds, however, result from an interaction between both components; such studies are still lacking from the literature. In this paper, we investigate an integrated model of both concepts during tumor progression - the effects of tumor immunosurveillance and the consequences of a mutator phenotype of tumor cells. We introduce specific immune responses to a formulation of the quasispecies model and study the balance between evasion of immunosurveillance and prevention of an error catastrophe. This study reveals the effects of various tumor antigens on specific immune responses from the viewpoint of evolutionary dynamics, and provides new perspectives on optimum treatment strategies of tumors subjected to immunosurveillance and -editing.

## Results

During the early phases of tumorigenesis, immune system cells such as NK and CD8+ T cells attack tumor cells and may succeed in suppressing their expansion; this outcome is referred to as “tumor dormancy”. However, if the immune system cannot successfully eradicate a tumor, then eventually a subset of tumor cells will acquire the phenotypes necessary for immunoescape. Depending on the magnitude of the mutation rate of these cells, the tumor cell population may then be at risk of going extinct due to the generation of excess damage – the event of an error catastrophe. To investigate the dynamics, conditions, and likelihood of these events, we designed a mathematical model of tumor and immune system cells.

In the context of our mathematical model, initially there is only a single type of tumor cells – those cells that originally founded the tumor. Denote the abundance of these original tumor cells by 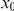. They divide at rate 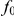 and die at rate 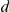. During each division of such a cell, a new variant tumor cell is produced with probability 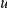. Each tumor variant may have evolved a phenotype which allows it to evade eradication by immune system cells. The different tumor variants are enumerated as cell types 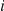, and the abundance of each type is given by 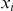. Let us first assume that all tumor variants divide at rate 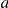 and die at rate 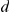. These assumptions ensure that intra-variant competition is stronger than inter-variant competition, such that each tumor cell only competes with cells of its own type for oxygen, nutrients, and space; these assumptions will be relaxed later on. The total number of tumor variants is denoted by 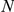. Since we consider a maximum number of 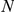 tumor variants, the growth rate of the original tumor cells reduces to 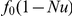.

**Figure 1:**
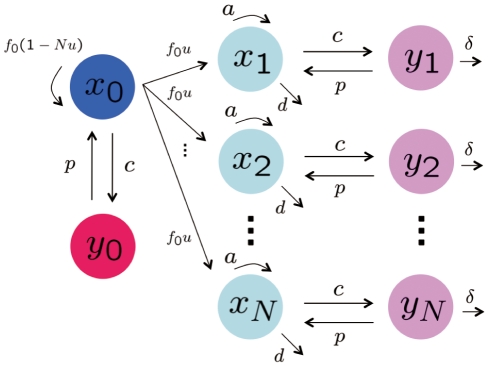
A mathematical framework of tumor cell evolution during immunosurveillance. The figure shows a schematic of the mathematical model. Initially, there is only a single type of tumor cells – those cells that originally founded the tumor. Their abundance is denoted by 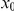; they divide at rate 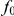 and die at rate 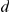. During each division of such a cell, a new variant tumor cell is produced with probability 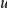. The tumor variants may have evolved a phenotype which allows them to evade eradication by immune system cells. The different tumor variants are enumerated as cell types 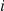, and their abundances are given by 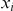. Tumor variants divide at rate 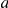 and die at rate 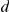. The total number of tumor variant types is denoted by 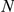. Since we consider 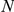 tumor variant types, the growth rate of the original tumor cells reduces to 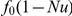. In addition to tumor cells, we also consider immune system cells that launch a specific immune response against each particular tumor variant. Denote the abundance of immune system cells specific to the original tumor clone by 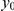, and those specific to tumor variant 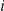 by 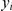 for 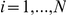. These immune system cells inhibit tumor variants at rate 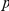 and are generated by interactions with the tumor cells at rate 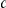. Immune system cells encounter tumor cells at a rate proportional to the latter cells' frequency, 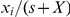; the parameter 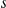 represents the coefficient of interactions between immune and tumor cells, and 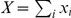 represents the total number of tumor cells. The lifespan of immune system cells is exponentially distributed with mean 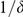 days.

In addition to tumor cells, we also consider immune system cells that launch a specific immune response against each particular tumor variant. Denote the abundance of immune system cells specific to the original tumor clone by 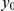, and the abundance of those specific to tumor variant 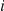 by 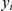, for 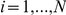. These immune system cells inhibit tumor variants at rate 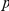 and are generated by interactions with the tumor cells at rate 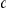. Figure 1 displays a schematic representation of this framework. Specific immune responses such as CTLs recognize their target tumor cells through random interactions and identification of antigens presented on the cell surface [42], [43]. In the context of the mathematical model, we assume that immune system cells encounter tumor cells at a rate proportional to the latter cells' frequency, 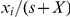; the parameter 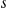 represents the coefficient of interactions between immune and tumor cells, and 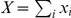 represents the total number of tumor cells. Expansion and differentiation of specific immune responses (e.g., precursor CTL proliferation and their differentiation into effector CTLs) are also regulated through interactions with tumor cells [44]. Therefore, immune system elimination of tumor cells and the proliferation of CTLs are described in a frequency-dependent way, with maximum elimination and proliferation rates of 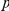 and 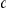, respectively. The lifespan of CTLs is considered to be exponentially distributed with mean 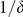 days.

### The basic mathematical model

With these considerations, we define the basic mathematical model including tumor variants and their specific immune responses by
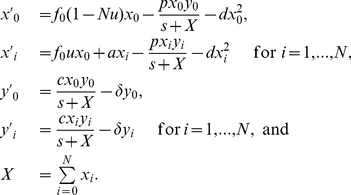
(1)


Baseline values of model parameters and their respective ranges used for simulations are presented in Table 1. A subset of these parameter values were estimated in [39], [45], [46]. Since the original tumor cell clone has been suggested to proliferate faster than variant tumor cells [37], [47], the division rate of variant tumor cells is 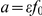 where 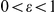; we assume a default value of 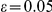 but also perform sensitivity analyses (see a later section). We found that, although some of these values are rough estimates and might deviate when measured by other groups or in other systems, our main results are qualitatively preserved within broad ranges around our baseline values.

**Table 1:** Baseline parameter values and their ranges for numerical simulations.

Parameter	Description (Units)	Value and Range	Reference
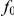	Fitness of original cancer cells ( 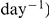	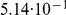	[39]
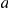	Fitness of variant cancer cells ( 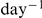 )	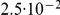	[37], [47]
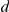	Degree of competition among variant cancer cells ( 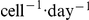 )	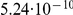	[39]
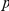	Maximum elimination rate of cancer cells ( 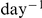 )	5.8	[39], [41]
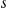	Coefficient of interaction between CTL and cancer cells	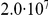	[39]
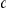	Maximum proliferation rate of CTLs ( 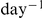 )	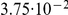	[39]
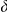	Decay rate of CTLs ( 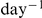 )	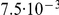	[46]
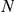	Number of cancer variants	[0,100]	-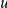	Mutation rate of original cancer cells	[0,0.05]	-

Let us now discuss the possible outcomes of interactions between the immune system and the tumor cell population: there may be tumor dormancy, partial immunoescape, complete immunoescape, and the event of an error catastrophe. In the dormancy state, immunosurveillance serves to effectively suppress the tumor cell population. In the partial immunoescape state, some tumor variants (but not all) achieve immunoescape while in the complete immunoescape state, the immune response is completely unsuccessful. Finally, in the error catastrophe state, the original tumor clone, which has the highest division rate, goes extinct due to the accumulation of excess alterations. We now outline how the original tumor clone, the tumor cell variants, and the specific immune system cells behave during the accumulation of alterations and the evolution of higher mutation rates.

### Tumor immunoescape and error catastrophe

**Figure 2:**
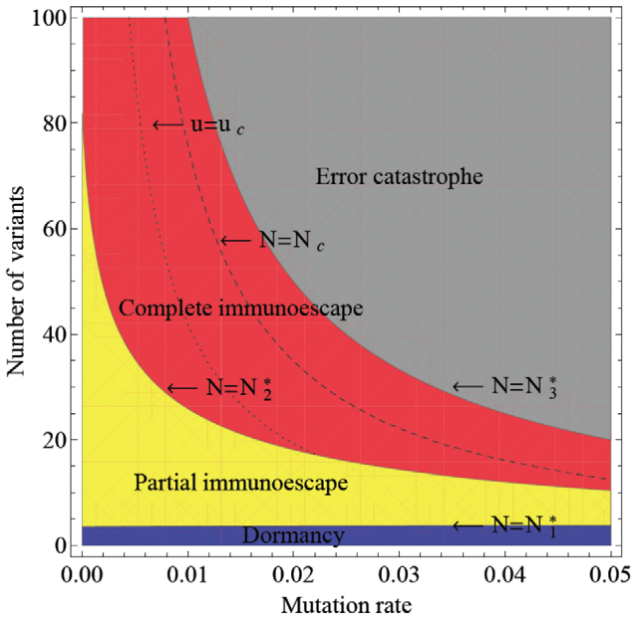
The steady-state regime of tumor immunoescape and error catastrophe. The figure displays the effects of the mutation rate (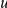) and the maximum number of tumor variant types (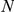) on the evolutionary dynamics of tumor cells. There are three thresholds that determine the outcomes of interactions between immune system cells and tumor cells. When the maximum number of tumor variant types is less than 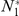, then immune responses suppress all tumor cells (blue), but if the variant number exceeds this threshold, then tumor cells are able to escape from their specific immune responses (yellow). Once the number of tumor variants exceeds 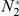, all tumor cells completely escape from immune responses (red). However, if their number exceeds 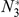, then an error catastrophe occurs (gray) in which original tumor cells cannot maintain a functioning genome due to excess error. Note that as the mutation rate increases, the threshold 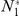 increases but 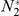 and 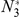 decrease. In situations of complete immunoescape (red), we obtain two thresholds regarding the total number of tumor cells, 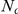 and 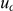. The total number of tumor cells increases until the number of the variants and the mutation rate, respectively, exceed 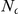 (dashed line) and 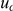 (dotted line), but then decreases after passing these thresholds.

The four qualitative outcomes of the interaction between tumor cells and the immune system – dormancy, partial and complete immunoescape as well as error catastrophe – are most significantly influenced by two systems parameters: the mutation rate generating tumor variants (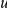) and the maximum number of tumor variant types that can emerge (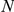). We therefore investigated the dynamics of tumor evolution in dependence of these parameters, and identified three analytical thresholds (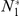, 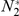, and 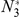) separating the potential outcomes (Figure 2). The formulas and detailed mathematical analyses of these thresholds are provided in the Methods section.

As long as the number of tumor variants is less than the first threshold, 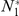, immune responses suppress all tumor variants (tumor dormancy). When the number of variants exceeds this threshold, however, then some tumor cells escape from the specific immune response (partial immunoescape). Once the number of variants passes the second threshold, 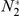, all tumor cells escape from immune responses (complete immunoescape). This finding implies that tumor cells can evade immune surveillance by accumulating a sufficiently large extent of intratumor heterogeneity. However, if the number of variants exceeds the third threshold, 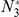, then an error catastrophe of tumor cells occurs, in which the original tumor clone can no longer maintain an expanding population and the original tumor cells therefore go extinct. We also found that, as the mutation increases, the threshold 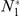 increases while 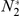 and 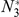 decrease. In all scenarios, however, tumor eradication is unlikely – although the tumor cell burden may shrink by a large amount – when the growth rate of the original tumor clone is negligibly small as compared to their death rate by apoptosis and/or interactions with the immune system.

We have thus established that although high rates of accumulating alterations allow tumor cells to reach a state of complete immunoescape, those cells with an excessively high mutation rate suffer an error catastrophe as the number of tumor variant types increases. These systems dynamics suggest that there is an optimum amount of instability that optimizes tumor evolution (i.e. maximizes the number of tumor cells) while maintaining a functioning genome.

### The optimal rate of tumor evolution

**Figure 3:**
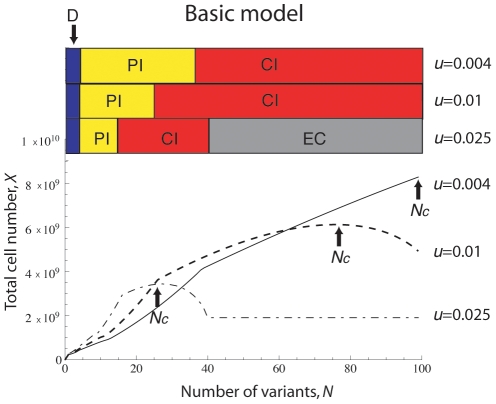
The total number of tumor cells during tumorigenesis. The figure displays the dynamics of the total number of tumor cells during the generation of an increasing number of tumor variants. In cases in which the dynamics are not stable, we show an average number of tumor cells. The abbreviations D, PI, CI, and EC represent the dormancy state, partial immunoescape state, complete immunoescape, and error catastrophe, respectively. The solid, dashed, and dash-dotted lines, respectively, represent mutation rates of 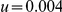, 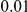 and 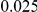. The total number of tumor cells decreases once the number of tumor variant types, 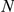, exceeds a threshold 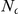 (for 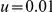 and 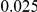) and eventually suffers an error catastrophe as soon as the variant number exceeds 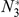 (for 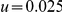). Tumor cells with high mutation rates increase in abundance by accumulating a large number of tumor variant types during early phases of tumor progression, but are incapable of efficient expansion in later phases due to the occurrence of an error catastrophe.

Let us now investigate the system dynamics for varying mutation rates and identify those regimes in which the total tumor cell number is maximized. Every time a new tumor variant arises, the dynamics of tumor evolution rapidly converges to its steady state; we therefore analyze the dynamics in steady state. The total number of tumor cells depends on the number of variants as well as the mutation rate, and an optimum combination of these parameter values exists that maximizes the total tumor cell number. In Figure 3, we demonstrate how the total number of tumor cells is affected by the number of tumor variant types for three different cases in which the mutation rate is 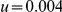, 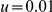, and 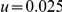, respectively. Detailed mathematical analyses of those equilibria are provided in the Methods section.

In situations in which all tumor cells are effectively suppressed by the immune response (tumor dormancy), the total number of tumor cells increases with the number of variant types. In situations in which some tumor cell types manage to escape from immune surveillance, the total number of tumor cells increases as both the number of variant types and the mutation rate increase (Figure 3). However, in situations in which all tumor cell types completely escape from their specific immune responses, there exist two thresholds regarding the total number of tumor cells: 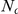 and 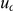 (see Figure 2). In this scenario, the total number of tumor cells increases until the number of variant types and the mutation rate, respectively, exceed the values of 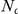 and 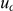; once crossing these thresholds, the tumor cell number decreases as 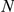 and 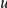 further increase. Therefore, tumor cells with an excessively high mutation rate cannot continue to become more abundant as the number of variant types increases (Figure 3), but there is an optimum, non-trivial parameter regime that maximizes the number of tumor cells.

Our results demonstrate that there are two strategies to maximize the rate of tumor evolution so that the total tumor cell mass is maximally large: one is to maintain a low mutation rate, since then the tumor cell population can increase the number of variant types along the threshold 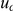 (see Figure 2); another is to keep the number of variant types relatively small, since then the tumor cell population can increase the mutation rate along the threshold 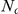 (see Figure 2). When both the mutation rate and the number of variant types are large, then the tumor cell population cannot maintain its maximum number without decreasing one of the two parameters.

### Fitness of variant tumor cells

Let us now investigate how the division rate of variant tumor cells affects the evolution of tumor cells during their interaction with immune system cells. Recall that in the basic model, the division rate is 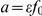, and that the threshold for an error catastrophe to occur (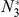) is independent of the division rate. To investigate the dependence of the system behavior on this division rate, we chose four different 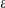 and performed a sensitivity analysis for the thresholds 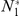 and 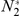.

**Figure 4:**
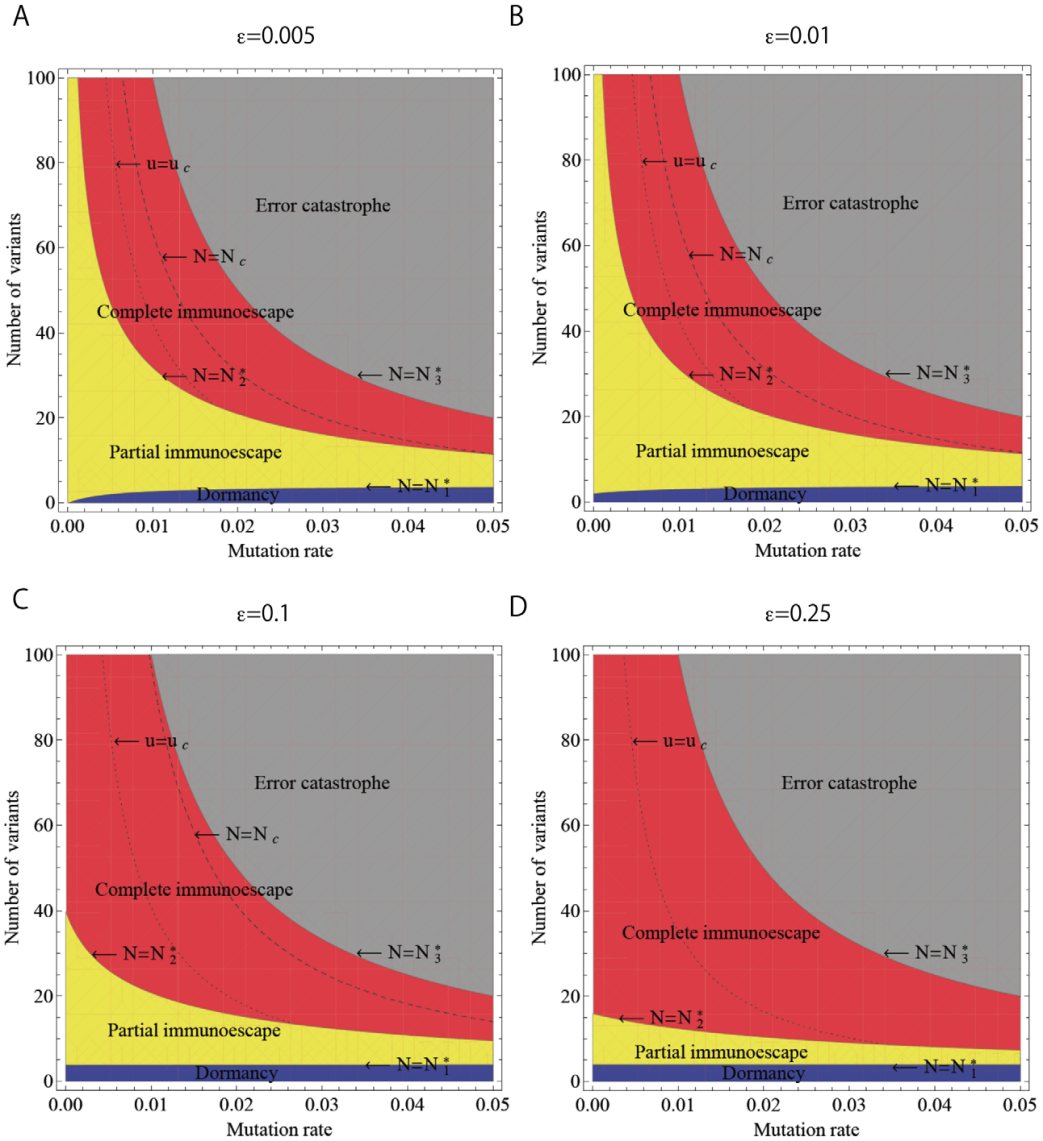
The effects of change in the fitness of variant tumor cells. The figure displays how the fitness of variant tumor cells, 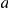, affects the dynamics of interactions between tumor and immune system cells. Parameters are (A) 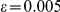, (B) 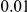, (C) 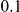, and (D) 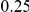. As the fitness of variant tumor cells increases, the parameter regime in which immunoescape is possible becomes larger; however, the qualitative behavior of the system dynamics is preserved.

Figure 4 displays how the division rate of variant tumor cells influences the outcome of tumor immunoescape in the plane of mutation rates (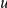) and the number of tumor variants (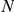). The four panels of the figure represent cases with different values of 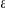. The higher the fitness of variant tumor cells becomes, the more easily they escape from immunosurveillance. However, the qualitative profiles of the system dynamics are preserved; that is, tumor cells with high mutation rates tend to reach a complete immunoescape while tumor cells with low mutation rates effectively produce a diverse population and thus increase in number.

### Extensions of the mathematical model

Let us next consider additional effects arising during tumor progression such as competition among tumor cells of different variant types, the presence of an innate immune response such as NK cells, which non-specifically target all tumor variants, and differential growth rates among tumor cell variants. In order to investigate the conditions for outcomes such as tumor immunoescape and error catastrophe in these more complex scenarios, we established an extended model, given by
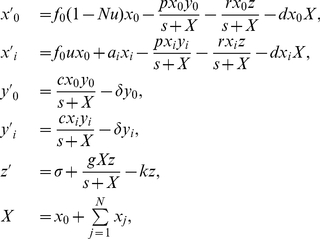
(2)where 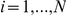. The parameter 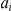 represents the division rate of tumor variant 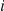. We now assume that each tumor cell competes with all other tumor cells so that the death term becomes 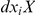. Furthermore, the variable 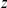 describes innate immune responses, for instance by NK cells which attack tumor cells without antigen specificity. The parameters 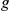, 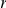, 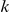, and 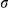 represent, respectively, the maximum proliferation rate of NK cells, the maximum elimination rate of tumor cells by NK cells, the decay rate of NK cells, and a constant source of NK cells.

**Figure 5:**
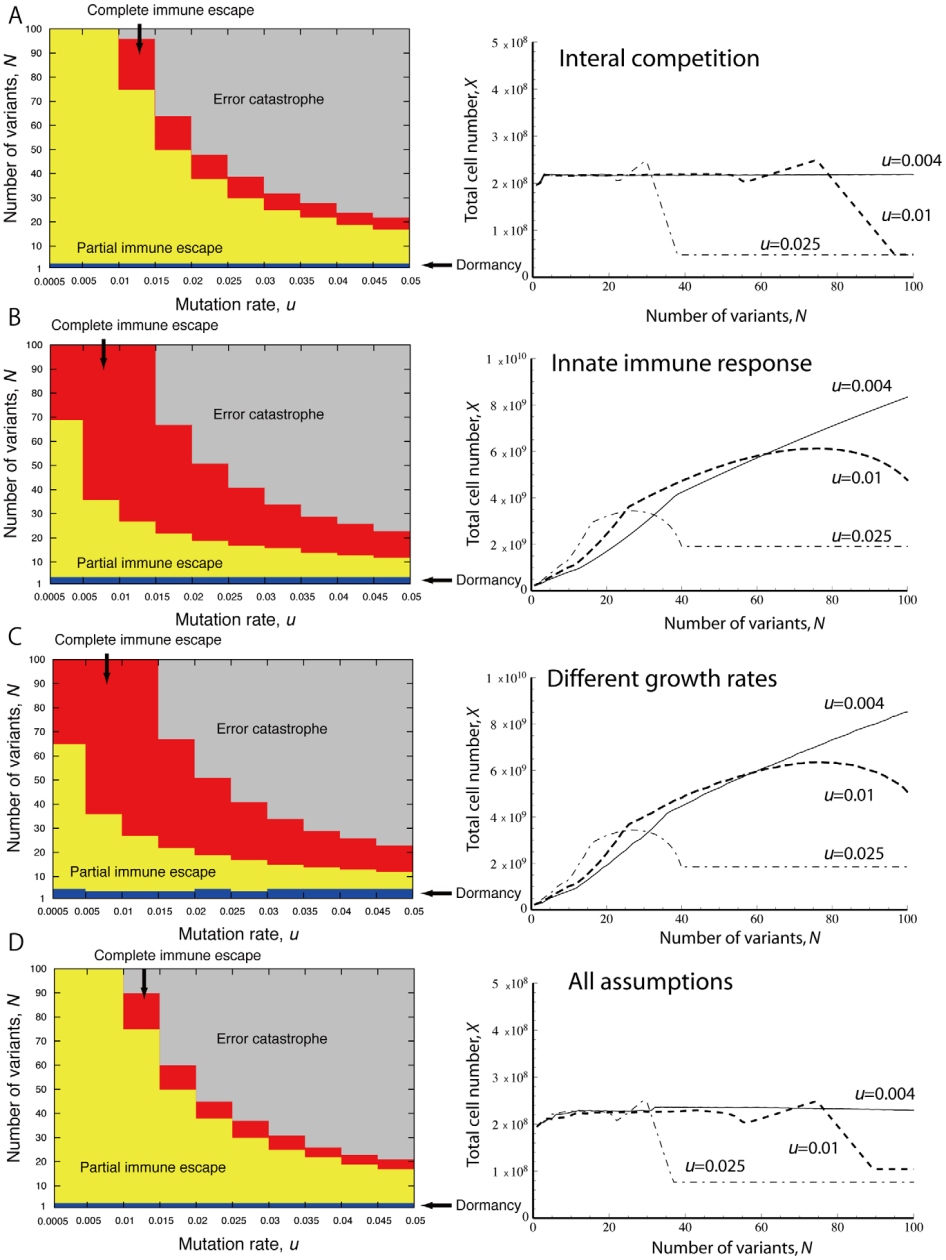
More complex scenarios arising during tumor progression. The figure displays the effects of more complex situations arising during tumorigenesis such as internal competition between tumor cells of different variant types (A), the presence of an innate immune response such as NK cells, which inhibit all tumor cells equally (B), different growth rates of tumor variants (C), and all of the above (D). These factors are incorporated into the basic model, equation (1). The qualitative behavior of the system is preserved although the size and identity of the parameter regimes for various outcomes are different. Additional parameters in the extended models are (B) 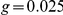, 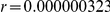, 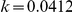 and 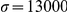, (C) variant growth rates 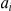 are randomly sampled from a normal distribution with mean = 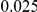 and variance = 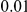, and (D) all of the above.

The dynamics of tumor progression considering these situations are shown in Figure 5. We investigated how inter-variant tumor cell competition (Figure 5A), incorporation of an innate immune response (Figure 5B), growth rates which differ between individual tumor variants (Figure 5C), and all three effects simultaneously modulate the thresholds between outcomes as well as the optimum parameter regimes for maximizing tumor cell numbers. Competition among tumor cells of the same variant type renders it difficult for the tumor cell population to completely escape from immune surveillance and to increase the total cell number beyond a relatively small value, irrespective of the mutation rate (Figure 5A). However, when only an innate immune response is present without inter-variant competition, then there is a larger parameter regime in which complete immunoescape is possible. Furthermore, the total number of tumor cells is larger in this situation as compared to the above case (Figure 5B). Similar to this scenario, the presence of different growth rates for individual variant clones allows for the existence of a large number of tumor cells as well as a large regime in which complete immune escape can be achieved (Figure 5C). Finally, when all three aspects are combined in the mathematical model, then the region of complete immune escape becomes very small; this effect is mainly driven by the incorporation of interal competition. The total number of tumor cells also remains below a rather small threshold for this case (Figure 5D).

### Optimum treatment strategies for diverse tumor cell populations

Finally, let us discuss the effects of different treatment modalities on the rates of cancer progression and the chance of immunoescape. Since the behavior of tumor cells and thus patient outcomes are to a considerable extent driven by the interactions between tumor and immune system cells, we considered both traditional chemotherapy and treatment options that stimulate the immune system to launch or sustain an attack against the tumor cell population. In general, immune therapies have not been proven to be very effective against many tumor types; one of the few exceptions is represented by adoptive cell therapy, which is used in the treatment of metastatic melanoma and causes regressions in about 50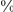 of patients [48]. Recently, however, synergistic effects of immunotherapy in combination with chemotherapy have been reported in both human and animal trials [49], [50], [51], and several mechanisms were identified that may explain these synergistic effects [52].

**Figure 6:**
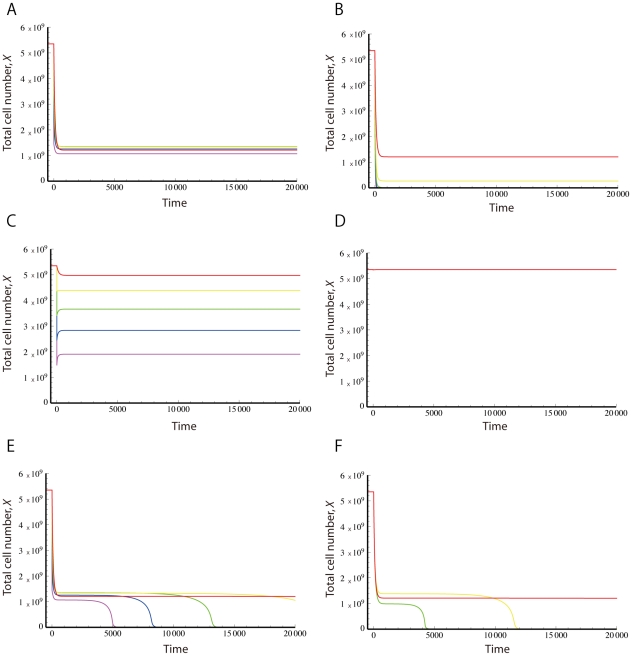
Optimal treatment strategies. The figure displays the effects of several treatment modalities on the total tumor burden. Treatment starts at time 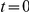. The number of tumor variant types and the mutation rate are given by 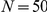 and 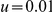. We consider situations in which tumor cell populations have reached the state of complete immunoescape before the initiation of therapy. (A and B) Chemotherapy is administered which reduces the number of tumor variant types and induces tumor cell deaths proportional to the tumor cell number. (C) Chemotherapy is administered which reduces the number of variant types and the growth rates of tumor cells. (D) Immunotherapy is used which replenishes the number of tumor-specific immune cells. (E and F) Chemotherapy and immunotherapy are administered which reduce the number of tumor variant types, induce tumor cell deaths, and replenish specific immune cells. Parameters are (A) 10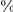 of tumor cells are killed per time unit by chemotherapy (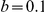) and the number of tumor variants decreases to 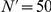 (red), 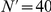 (yellow), 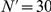 (green), 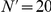 (blue), and 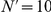 (purple); (B) the number of tumor variants does not change (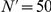), but 10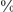 (red), 20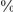 (yellow), 30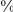 (green), 40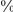 (blue), and 50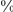 (purple) of tumor cells are killed by chemotherapy; (C) growth rates are reduced by 10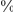 by chemotherapy and the number of tumor variants decreases to 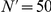 (red), 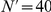 (yellow), 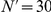 (green), 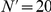 (blue), and 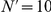 (purple); (D) 1 (red), 100 (yellow), and 10,000 (green) immune cells specific to each tumor variant are added into the system by the administration of immunotherapy; (E) 10,000 immune cells specific to each tumor variant are added into the system by immunotherapy, 10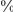 of tumor cells are killed per time unit by chemotherapy, and the number of tumor variants decreases to 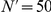 (red), 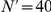 (yellow), 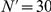 (green), 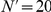 (blue), and 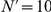 (purple) by chemotherapy; and (F) 10000 immune cells specific to each tumor variant are added into the system by the administration of immunotherapy, 10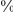 of tumor cells are killed per time unit by chemotherapy, the number of tumor variants does not change (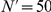), and mutation rates are 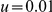 (red), 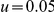 (yellow), and 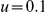 (green).

To study the effects of chemotherapy, immune therapy, and combination therapy on the dynamics of tumor evolution, we introduced a series of different treatment types into the mathematical framework and identified optimal treatment strategies for diverse tumor cell populations (Figure 6). These different treatment modalities were tested in situations in which tumor cells had previously achieved complete immunoescape and consisted of a large number of tumor cells. The number of tumor variants and the mutation rate were considered to be 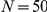 and 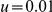 at the time of treatment initiation. Chemotherapy then reduces the number of tumor variants and kills tumor cells proportional to the tumor cell number present. We also considered the case in which chemotherapy reduces the growth rate of tumor cells. Then the model after treatment initiation is given by
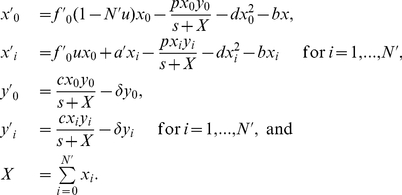
(3)Here chemotherapy reduces the number of tumor variants to 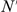 and either kills the tumor cells at rate 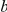 or reduces the growth rates to 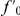 and 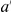. Immunotherapy increases the number of specific immune cells (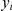 for 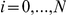) during treatment.

We then utilized this system to investigate optimum treatment strategies. First, let us consider the effects of chemotherapeutic agents which reduce the number of tumor cells by inducing cell deaths at a rate proportional to the cell number present within the tumor. Administration of such treatments decreases the total cell number, but may not be capable of leading to complete eradication of all tumor cells (Figure 6A) unless its effects are sufficiently (and maybe unrealistically) strong (Figure 6B). Second, consider chemotherapeutic drugs which reduce the number of tumor variant types as well as the growth rates of tumor cells. Again, administration of such treatments decreases the total cell number but is incapable of achieving complete eradication of tumor cells (Figure 6C). Third, consider the administration of immunotherapy which increases the population of tumor-specific immune system cells. Such therapy alone is not able to decrease the abundance of tumor cells by a large extent (Figure 6D). However, when combining chemotherapy and immunotherapy, an effective decrease of the tumor cell population can be achieved, which may ultimately lead to tumor eradication and a cure (Figure 6E). Notably, in situations in which the mutation rate is small, the administration of combination therapy is more successful in eradicating all tumor cells as compared to situations in which the mutation rate is high (Figure 6F).

In conclusion, our mathematical model predicts successful outcomes of combination therapy when (i) chemotherapy is administered which induces tumor cell death at a significantly large rate, or (ii) combination therapy is administered which reduces the number of tumor variants, induces tumor cell death, and replenishes immune cell populations. When the mutation rate of tumor cells is small, combination therapy is more effective than when variations arise at a large rate. An explanation of these findings can be found in Figure 2 – activation of the immune response alone does not change the state of the tumor cell population once it has reached complete immunoescape; in that case, the number of tumor cells does not decrease (Figure 6D). A reduction of the number of tumor variant types and tumor cells by administering chemotherapy alone allows for partial immunoescape or dormancy states, but there is an insufficient abundance of immune system cells to effectively control the tumor cell population (Figure 6A). However, combination therapy which enables immune cells to be activated in the states of partial immunoescape or dormancy is capable of eradicating the tumor (Figure 6E). Thus, our mathematical framework is capable of identifying those treatment modalities that have the potential to lead to a cure of the tumor.

## Discussion

In this paper, we have investigated the dynamics of tumor progression under immune system surveillance while considering the effects of increasing rates at which (epi)genetic alterations are generated. We defined specific situations that can arise due to the interactions of immune system cells and tumor cells. When the tumor cell population is able to persist under immunosurveillance without leading to tumor growth, then a state of tumor dormancy ensues. Should the immune system not be capable of efficiently suppressing the tumor cell population, then partial or complete immunoescape is possible, depending on whether some or all tumor clones evade immune system inhibition. Finally, an error catastrophe occurs when the tumor cells evolve mutation rates that are incompatible with the maintenance of a functioning genome due to excess error.

The dynamics of the system and likelihood of these different states depend on the rate at which variability emerges in the population (denoted by the mutation rate 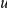 per cell division) as well as the number of distinct tumor clones (given by 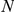) that are distinguished by their capabilities of generating a specific immune response (see Figure 2). If both quantities are excessively large, then an error catastrophe occurs and the original tumor cell population goes extinct. In intermediate regimes, states of dormancy and partial or complete immunoescape are possible. We also investigated the extent to which the total number of tumor cells depends on these parameters and identified regimes in which the maximum number of tumor cells is attained. Moreover, we relaxed the model assumptions to consider more complex scenarios such as growth competition among tumor variants, innate immune responses that non-specifically recognize and kill tumor cells, and different growth rates of tumor variants. These studies revealed that the patterns of states do not vary significantly as the assumptions of competition, growth, and innate immune responses are altered; however, internal competition among tumor variants renders it difficult for tumor cells to achieve complete immune escape.

Finally, we investigated the effects of different treatment modalities on the rates of tumor progression and found that administration of both chemotherapy and immunotherapy leads to optimum response rates, thereby confirming recent experimental findings [49], [50], [51]. These investigations have direct implications for the clinical management of cancers since they incorporate both mutator phenotype and the interactions between tumor cells and the immune system. A consideration of these factors is essential for an understanding of the dynamics of tumor cell populations evolving during immune system attacks. Our results thus suggest that combination therapy incorporating both chemotherapeutic and immunostimulatory agents would lead to best results in the clinic.

Our mathematical framework represents only one possibility of modeling the system of tumor and immune system cells. This modeling choice was made for reasons of mathematical simplicity as well as availability of parameter estimates; however, several model extensions are conceivable. For instance, the spatial components of the system could be incorporated such that the spatio-temporal aspects can be considered. Also, we have neglected stochasticity in our formulation of the mathematical model since both population sizes and mutation rates are large, and therefore deterministic dynamics dominate. However, for more detailed investigations – such as a determination of the probability that a certain phenotype arises – the stochasticity of the system cannot be neglected. Such studies will be the topic of future contributions. Furthermore, interactions with the microenvironment such as with stromal cells and other factors could be considered. The incorporation of these extensions are complicated by the fact that few quantitative estimates are available. The determination of system parameters necessary for including such factors into a mathematical framework is an important goal of the field.

## Methods

### Mathematical analysis

Let us first consider the basic model, equation (1), in detail. The basic model can be considered qualitatively as a 4-dimensional ODE system (although equation (1) is a 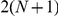-dimensional ODE system) when analyzing the equilibria, since all parameters in the equations describing 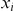 and 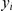 are the same, and therefore 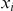 and 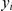 have the same properties at the equilibria. Hence we can consider that 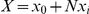 with respect to the equilibria, where 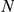 is a model parameter.

### Existence conditions of equilibria

We investigated the existence conditions of the equilibria of model (1). The model has seven possible equilibria:
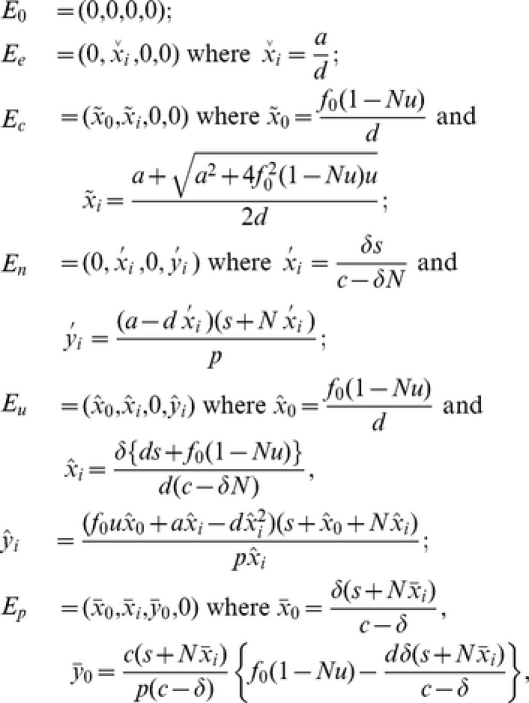
and 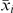 is the positive root of the following equation:
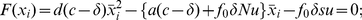


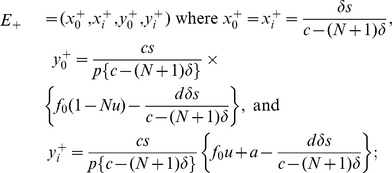



While the equilibria 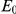 and 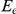 always exist, 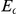 only exists if 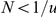. Furthermore, 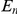 exists if 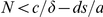, and 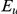 exists if 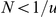, 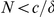 and 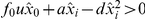 because 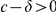. The existence condition of 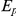 is 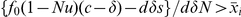. In addition, we calculate the existence conditions of 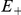 as follows. Note that 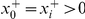 and 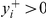 are equivalent to 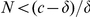 and 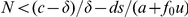, respectively. If 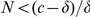, then we have the following relations:
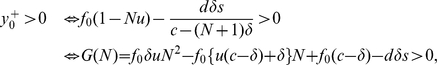
because 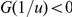, 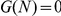 always has real roots 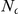 and 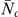 (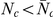). Here we assume that 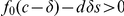: otherwise 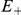 never exists and 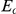 becomes stable. Therefore, 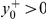 is equivalent to 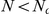. Note that the roots of 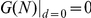 are 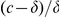 and 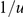, which implies that 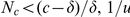. Furthermore, when 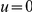, 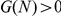 is equivalent to 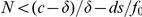. Since 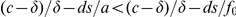 in the context of 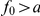 (which is a suitable assumption), we can roughly estimate that 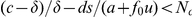 if 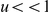. Consequently, if 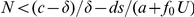, 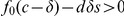, 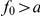 and 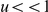, then 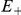 exists.

### Derivation of thresholds

Consider the situation in which 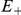 exists, all tumor cells are suppressed by their specific immune responses, and the number of tumor variants is 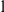 (i.e., 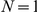). Let us investigate the transversal eigenvalue in the 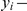
direction at 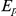. Here we define
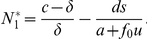
Since 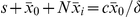, the transversal eigenvalue is evaluated as follows:
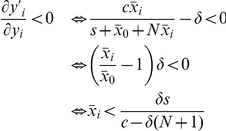


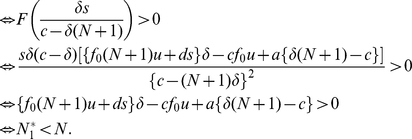
This result implies that, if 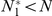, then 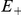 disappears from 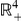 and 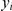 approaches 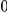 near 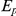. Hence only the original tumor cells are suppressed by their specific immune responses while tumor variants escape from immune surveillance once the number of tumor variant exceeds 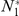. Next, we investigate the transversal eigenvalue in the 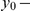
direction at 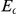. Here we define 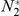 to satisfy
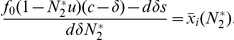
The transversal eigenvalue is evaluated as follows:
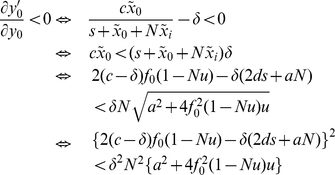


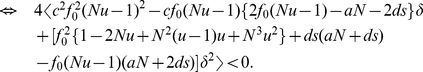
On the other hand, we have the following relations:
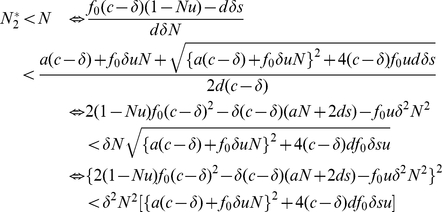


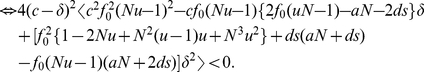



This result implies that 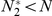 is equivalent to 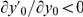 near 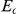; that is, if 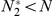, then 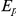 disappears from 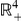 and 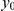 converges to 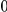 near 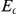 (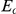 is stable). Thus, the immune response against the original tumor clone also becomes inactivated if the number of tumor variants exceeds 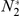. Note that we consider a restricted region of 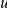 and 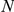, where 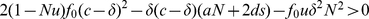. Consequently, all tumor cells escape from their immune responses once the number of tumor variants exceeds 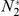. Furthermore, the original tumor cell clone is no longer sustainable (i.e., an error catastrophe occurs) as soon as the number of the variants exceeds 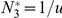.

### The total number of tumor cells

Let us now calculate the total number of tumor cells at equilibrium. Note that the dynamics of the basic model, equation (1), might not converge to an equilibrium, but may oscillate if 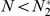.

When the number of tumor variants is 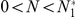, then the total number of tumor cells at 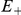 is 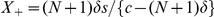. Since we evaluate 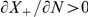 and 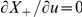, the total number of tumor cells increases as the number of variants grows. When the number of variants is 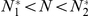, then the total number of tumor cells at 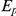 is 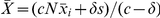. Again, as we evaluate 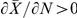 and 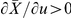, the total number increases as the number of variants and the mutation rate increase. When the number of variants is 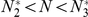, then the total number of tumor cells at 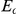 is 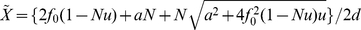. Here we find critical thresholds 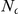 and 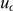 such as 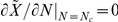 and 
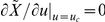
. Therefore, the total number of tumor cells increases as long as the number of variants and the mutation rate are 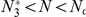 and 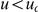, respectively. However, once the number of variants and the mutation rate exceed 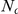 and 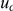, respectively, the total number of tumor cells decreases. Eventually, when the number of variants is 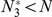, the total number of tumor cells at 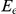 is 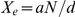.
